# Volume of Hypothalamus as a Diagnostic Biomarker of Chronic Migraine

**DOI:** 10.3389/fneur.2019.00606

**Published:** 2019-06-06

**Authors:** Zhiye Chen, Xiaoyan Chen, Mengqi Liu, Lin Ma, Shengyuan Yu

**Affiliations:** ^1^Department of Radiology, Chinese PLA General Hospital, Beijing, China; ^2^Department of Neurology, Chinese PLA General Hospital, Beijing, China; ^3^Department of Radiology, Hainan Hospital of Chinese PLA General Hospital, Sanya, China

**Keywords:** hypothalamus, episodic migraine, chronic migraine, chronicification, volume, voxel-based morphomethy, functional connectivity

## Abstract

It is believed than hypothalamus (HTH) might be involved in generation of migraine, and evidence from high resolution fMRI reported that the more anterior part of HTH seemed to play an important role in migraine chronification. The current study was aimed to identify the alteration of morphology and resting-state functional connectivity (FC) of the hypothalamus (HTH) in interictal episodic migraine (EM) and chronic migraine (CM). High-resolution structural and resting-state functional magnetic resonance images were acquired in 18 EM patients, 16 CM patients, and 21 normal controls (NC). The volume of HTH was calculated and voxel-based morphometry (VBM) was performed over the whole HTH. Receiver operating characteristics (ROC) curve analysis was applied to evaluate the diagnostic efficacy of HTH volume. Correlation analyses with clinical variables were performed and FC maps were generated for positive HTH regions according to VBM comparison. The volume of the HTH significantly decreased in both EM and CM patients compared with NC. The cut-off volume of HTH as 1.429 ml had a good diagnostic accuracy for CM with sensitivity of 81.25% and specificity of 100%. VBM analyses identified volume reduction of posterior HTH in EM vs. NC which was negatively correlated with headache frequency. The posterior HTH presented decreased FC with the left inferior temporal gyrus (Brodmann area 20) in EM. Decreased volume of anterior HTH was identified in CM vs. NC and CM vs. EM which was positively correlated with headache frequency in CM. The anterior HTH presented increased FC with the right anterior orbital gyrus (AOrG) (Brodmann area 11) in CM compared with NC and increased FC with the right medial orbital gyrus (MOrG) (Brodmann area 11) in CM compared with EM. Our study provided evidence of structural plasticity and FC changes of HTH in the pathogensis of migraine generation and chronification, supporting potential therapeutic target toward the HTH and its peptide.

## Introduction

Migraine is a common disabling primary headache disorder characterized by multiphase attacks of headache and a number of accompanying symptoms. Chronic migraine(CM) is defined as headache occurring on ≥15 days/month for more than 3 months with migraine features on ≥8 days/month (1). CM affects approximately 2% of the adult population in western countries, imposing substantial burdens on individual sufferers, their families and society. Although this disorder is highly disabling and prevalent, it remains largely underdiagnosed and undertreated (2). The chronification occurs in about 2.5% or more of episodic cases annually (3, 4). A number of potential risk factors may be associated with the transition to CM (3). But the underlying pathophysiologic mechanisms leading to migraine chronification are still unknown.

Neuroimaging has played a significant role in the current understanding of pathophysiologic processes behind migraine. The processes of migraine attacks seem to lead to increased sensitivity or hyperexcitability of different brain regions, facilitating occurrence of headache and aura (5). The hypersensitivity of attack-generating brain regions may lead to the enhanced susceptibility to attack generation. Recent evidences suggested that the brainstem, central dopaminergic system and hypothalamus (HTH) might be crucially involved in generation of migraine attack (5). The HTH has multiple functions in maintaining homeostasis by controlling the endocrine system, coordinating the activity of the sympathetic and parasympathetic nervous systems, integrating psyche and soma, regulating circadian rhythms and arousal, and in nociceptive processing (6). Some clinical features of migraine point toward HTH involvement, such as yawning, tiredness and mood changes in the premonitory phase (7), the circadian rhythmicity of attacks (8) and the association of attacks with hormonal status and the menstrual cycle (9). Neuroimaging studies revealed activation of HTH and altered functional coupling with the spinal trigeminal nuclei and the region of the migraine generator, i.e., the dorsal rostral pons in the premonitory and acute pain phase of migraine, suggesting that the real driver of migraine attacks might be the functional changes in hypothalamo-brainstem connectivity (10–13). In addition, a recent study found that the more anterior part of HTH seemed to play an important role in migraine chronification (14). Therefore, the HTH might be an important biomarker for the diagnosis and treatment of migraine.

As far as we know, there is no study focusing on morphometric analysis on the HTH in chronic migraine. Structural neuroimaging may provide an easier way than functional neuroimaging in clinical practice and add complementary information in speculating mechanisms of the HTH in the pathogenesis of migraine. We had the following hypothesis: (1) the volume of HTH may change in migraineurs; (2) the volume of HTH sub-regions may change in different patterns for CM and interictal episodic migraine (EM); (3) there might be altered functional connectivity(FC) of the positive sub-regions in HTH. To address these hypotheses, we prospectively conducted a study investigating the morphological changes and FC of the HTH in patients with CM, EM and healthy controls (HC) via high-resolution structural and functional magnetic resonance imaging (MRI) by calculating the volume of HTH, performing voxel-based morphometry (VBM) analysis over the whole HTH, and generating FC maps. By doing this, we aimed to investigate the role and possible mechanisms of HTH in migraine and the chronification.

## Materials and Methods

### Subjects

Eighteen EMs, 16 CMs and 21 normal controls (NCs) were recruited from the International Headache Center, Department of Neurology, Chinese PLA General Hospital. All the patients should fulfill the International Classification of Headache Disorders, 3rd Edition (ICHD-III) criteria of episodic or chronic migraine without aura (1), and the inclusion criteria was as follows: (1)The diagnosis of migraine refers to 1.1 Migraine without aura; (2) EM is defined as migraine attack days being <15 days per month, and diagnosis of CM refers to 1.3 CM in ICHD-III; (3) no migraine preventive medication used in the past 3 months; (4) absence of any chronic disorders, including hypertension, diabetes mellitus, cardiovascular diseases, cerebrovascular disorders, neoplastic diseases, other subtypes of headache, chronic pain other than headache, severe anxiety or depression preceding the onset of headache, psychiatric diseases, etc.; (5) absence of alcohol, nicotine, or other substance abuse. The inclusion criteria of NC were similar to those of patients, except for the first three items. NC should never have any primary headache disorders or other types of headache in the past year. The exclusion criteria were the following: cranium trauma, illness interfering with central nervous system function, psychotic disorder, and regular use of a psychoactive or hormone medication.

All the patients were given with the Visual Analog Scale (VAS) for the pain intensity evaluation, the Migraine Disability Assessment Scale (MIDAS), Hamilton Anxiety Scale (HAMA) for the anxiety evaluation, Hamilton Depression Scale (HAMD) for the depression evaluation and Montreal Cognitive Assessment (MoCA) for the cognitive function evaluation. All the subjects were right-handed and underwent conventional MRI examination to exclude the subjects with cerebral infarction, malacia, or occupying lesions. Alcohol, nicotine, caffeine, and other substances were avoided for at least 12 h before MRI examination. All the patients underwent MRI scanning at least 3 days after last migraine attack. CM patients may have headache but not with migraine feature during MRI scanning. The study protocols were approved by the Ethical Committee of Chinese PLA General Hospital and complied with the Declaration of Helsinki. Informed consents were obtained from all participants before the study.

### MRI Acquisition

All the MRI data were acquired on a GE 3.0T MR system (DISCOVERY MR750, GE Healthcare, Milwaukee, WI, USA) and a conventional eight-channel quadrature head coil was used. All subjects were instructed to lie in a supine position, and formed padding was used to limit head movement. High resolution structural images were acquired with a three-dimensional T1-weighted fast spoiled gradient recalled echo (3D T1-FSPGR) sequence [TR (repetition time) = 6.3 ms, TE (echo time) = 2.8 ms, flip angle = 15°, FOV (field of view) = 25.6 × 25.6 cm, Matrix = 256 × 256, NEX (number of acquisition) = 1]. Resting-state functional MR images were obtained using a gradient echo-planar imaging (EPI) sequence (TR = 2,000 ms, TE = 30 ms, flip angle = 90°, slice thickness = 3 mm, slice gap = 1 mm, FOV = 24 × 24 cm, Matrix = 64 × 64). One hundred and eighty axial EPI functional volumes were obtained over 6 min. Oblique axial T2-weighted imaging (T2WI), T1 fluid-attenuated inversion recovery (T1-FLAIR) and diffusion weighted imaging (DWI) were also acquired. All imaging protocols were identical for all subjects. No obvious structural damage and T2-visible lesion were observed based on the conventional MR images.

### MR Image Processing

**Figure 1:**
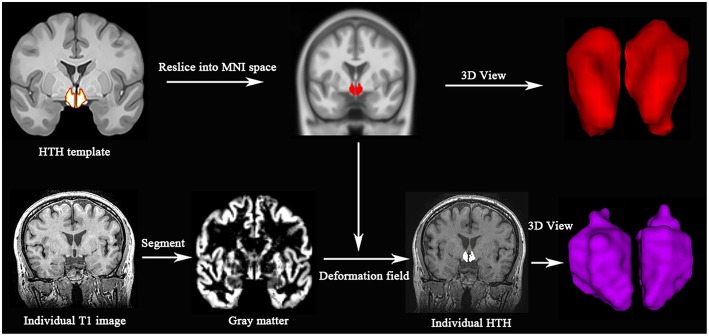
Individual HTH segment. Top line: high resolution probabilistic *in vivo* HTH template was resliced into MNI space; Bottom line: Individual T1 images were segmented, and generated deformation field, which would be used to generate individual HTH using pull-back strategy. Red and purple represent 3D visualization of HTH template and individual HTH.

All MR structural image data were analyzed with Statistical Parametric Mapping 12 (SPM 12) (http://www.fil.ion.ucl.ac.uk/spm/) running under MATLAB 7.6 (The Mathworks, Natick, MA, USA). The individual HTH segment included following steps: (1) Resliced a high-resolution probabilistic *in vivo* atlas of human HTH (15) into MNI space; (2) Individual structural images were performed with segment using DARTEL methods (16, 17), and generate gray matter, white matter and deformation field; (3) Individual HTH segment by applying the inverse deformation field to the HTH template; (4) Calculated the individual HTH volume by summing over all voxels in the individual HTH (Figure 1).

Voxel-based morphometry (VBM) analysis of HTH included following steps: (1) Gray matter images (generated by DARTEL segment) were smoothed with an isotropic 8 mm full width at half maximum Gaussian-kernel; (2) A general linear model was used to compare HTH volume changes on voxel level with age, sex and TIV as covariance; (3) Small volume correction (*P* < 0.05, no cluster size threshold) was applied in a region by the standard HTH template; (4) False discovery rate (FDR) was assessed to perform the multiple comparison corrections (*P* < 0.05)

**Figure 2:**
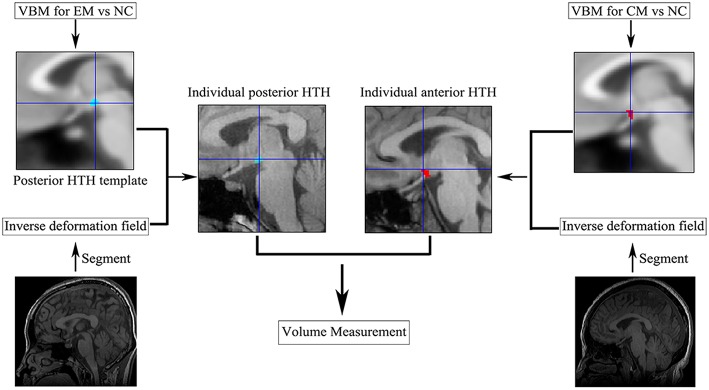
The individual anterior and posterior HTH segment. CM, chronic migraine; EM, episodic migraine; HTH, hypothalamus; NC, normal control; VBM, voxel-based morphometry.

The volume extraction of the altered volume of the HTH region in VBM processing (Figure 2): (1) All the positive clusters of VBM were saved as posterior HTH template for EM vs. NC comparison and anterior HTH template for CM vs. NC comparison, respectively; (2) Individual positive HTH segment by applying the inverse deformation field to the anterior and posterior HTH template to generate the individual anterior and posterior HTH; (3) Calculated the individual positive HTH volume by summing over all voxels in the individual positive HTH region.

The functional connectivity (FC) analysis was processed using DPABI software (V4.3_170105) (18) as following: (1) First 10 volumes of the resting-state functional images were removed; (2) Slice timing correction; (3) Head motion correction; (4) Structural images were coregistered to the functional images; (5) The nuisance regressors were removed according to Yan's methods (19), which included motion parameters and their derivatives, global, white matter, CSF time series; (6) The linear trend removal and temporal band-pass filtering (0.01–0.08 Hz); (7) Spatial normalization using by DARTEL; (8)The clusters were classified as the seeds based on VBM comparisons among EM, CM and NC; (9) FC was calculated and the individual FC maps were normalized to Z-maps using Fisher's Z transformation;(10) Smoothing with a 6 mm Gaussian kernel, and two-sample *t*-test was performed with age and sex as covariables to identify the brain regions with significant difference in FC with the positive HTH regions with altered volume among EM, CM, and NC. Significance was set as *P* < 0.001 without correction. The minimal number of continuous voxels was set based on the expected voxels per cluster. The statistical analysis was performed by SPM12 software.

### Statistical Analysis

The statistical analysis was performed by using IBM SPSS Statistics (version 23) and MedCalc (version 11.4.2.0). The normal distribution data presented by mean±standard deviation, and the non-normal distribution data presented by median (minimum, maximum). Age was performed with one-way analysis of variance, sex with Chi-square test, VAS with independent *t*-sample test, MIDAS, disease duration (DD) and headache frequency with Mann-Whitney U test because of non-normal distribution. HAMA, HAMD, and MoCA score were performed with one-way analysis with LSD method for variance homogeneity and Dennett's T3 method for variance non-homogeneity. HTH volume was performed with one-way analysis of covariance with age, sex, and total intracranial volume (TIV) as covariance. Pearson's correlation analysis was applied with the normal distribution data, and Spearman's method was applied with the non-normal distribution data. Significant difference was set at a *P* < 0.05. Receiver operating characteristics (ROC) curve analysis was applied to evaluate the diagnostic efficacy of HTH volume and area under the curve (AUC) was recognized reasonable diagnostic valuable with AUC set at >0.7.

## Results

### Comparison of Clinical Variables and HTH Volume Among NC, EM, and CM

Table 1 presented that there was significant difference for age between EM and CM, sex between NC and EM (*P* = 0.01 and 0.02, respectively). VAS showed no significance between EM and CM (*P* = 0.37). The disease duration showed no significant difference between EM and CM (*P* = 0.485). The headache frequency was significantly higher in CM than that in EM (*P* = 0.000). CM patients had a higher HAMA and HAMD score than that in EM (*P* = 0.003 and 0.033, respectively). MoCA score showed a significant difference among EM, CM and NC (*P* < 0.05). CM had a significantly higher MIDAS score compared with EM (*P* = 0.00).

**Table 1:** Comparison of clinical variables and HTH volume among NC, EM, and CM.

	**NC**	**EM**	**CM**	***F*-value**	***P*-value**
Age^a^ (year)	39.61 ± 10.09	33.39 ± 11.00	42.44 ± 8.65	3.69	0.03
Sex (F/M)^b^	18 (7/11)	18 (14/4)	16 (12/4)	7.20^c^	0.03
VAS	NA	8.33 ± 1.50	7.88 ± 1.45	0.90	0.37
DD (year)	NA	10 (0.5,30)^d^	9 (3,30)^d^	124^e^	0.485
HF (times/month)	NA	3 (1,10)^d^	30 (17,30)^d^	0^e^	0.000
HAMA^f^	10.33 (5,16)	15.67 ± 9.85	21.63 ± 10.98	7.334^g^	0.002
HAMD^h^	8.44 ± 4.13	10.89 ± 7.26	16.31 ± 10.52	4.672^g^	0.014
MoCA^i^	27.00 ± 2.52	30 (25,30)^d^	22.94 ± 5.37	14.238^g^	0.000
MIDAS	NA	12 (0,70)^d^	101.81 ± 13.49	8^e^	0.00
HTH (ml)^j^	1.58 ± 0.08	1.47 ± 0.12	1.38 ± 0.12	5.03	0.01

a *Significant difference presented between EM and CM (P = 0.01)*.

b *There is a significant difference between NC and EM (Chi-square value 5.00, P = 0.02)*.

c *Pearson Chi-square value*.

d *Median (minimum, maximum)*.

e *Mann-Whitney U value*.

f *CMhad a higher HAMA score than that in NC (P = 0.003)*.

g *post hoc multiple comparison using Dennett's T3 because of variance non-homogeneity*.

h *CM had a higher HAMD score than that in NC (P = 0.033)*.

i *There was a significant difference for MoCA score among EM, CM and NC (P < 0.05)*.

j *NC had a significant higher HTH volume than that of EM (P = 0.004) and CM (P = 0.031)*.

*NA, not available; VAS, Visual Analog Scale; MIDAS, Migraine Disability Assessment Scale; DD, disease duration; HF, headache frequency; HAMA, Hamilton Anxiety Scale; HAMD, Hamilton Depression Scale; MoCA, Montreal Cognitive Assessment*.

**Figure 3:**
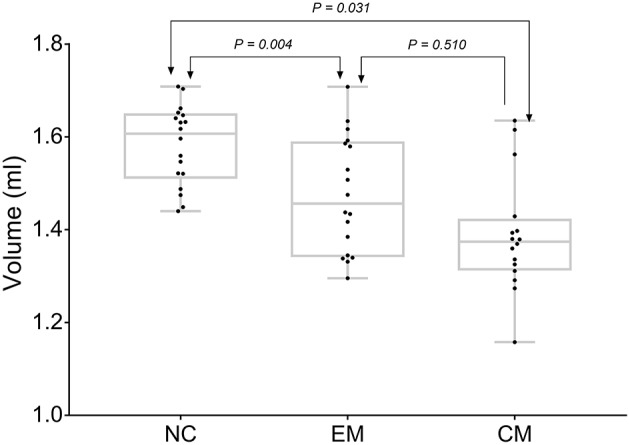
HTH volume distribution plot of NC, EM and CM patients.

CM had the lowest HTH volume (1.38 ± 0.12 ml) than that (1.58 ± 0.08 ml) of NC (*P* = 0.03). EM showed a decreased HTH volume (1.47 ± 0.12) compared with that of NC (*P* = 0.00). There was no significant difference between EM and CM (*P* = 0.51) (Figure 3).

### ROC Curve Analysis of HTH Volume Among NC, EM, and CM

The area under the receiver operating characteristic (ROC) curve (AUC) for NC vs. EM was 0.77 ± 0.08 (95% Confidence Interval 0.60 ~ 0.89), and the cut-off value was 1.437 with sensitivity 50%, specificity 100% and negative likelihood ratio 50%.

The AUC for NC vs. CM was 0.90 ± 0.06(95% Confidence Interval 0.75 ~ 0.98), and the cut-off value was 1.429 with sensitivity 81.25%, specificity 100% and negative likelihood ratio 19%.

**Figure 4:**
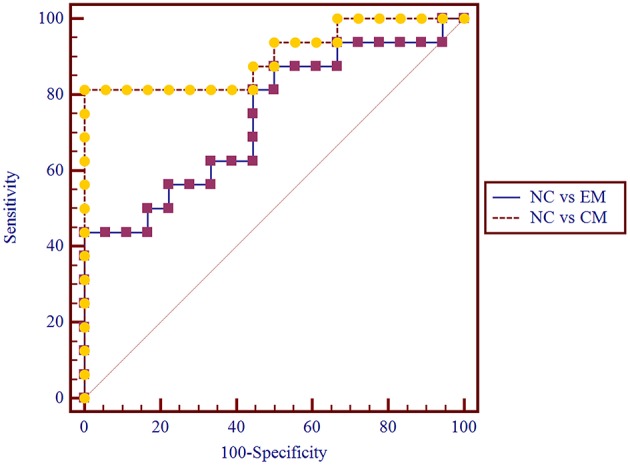
Pairwise comparison of ROC curve for NC vs. EM and NC vs. CM. The AUC for NC vs. CM was 0.90 ± 0.06, and for NC vs. EM was 0.77 ± 0.08.

Pairwise comparison of ROC curves between NC vs. EM and NC vs. CM confirmed that the difference between areas was 0.16 ± 0.09 (*P* = 0.08) (Figure 4).

### Voxel-Based Morphometry Analysis of HTH Among EM, CM, and NC

**Figure 5:**
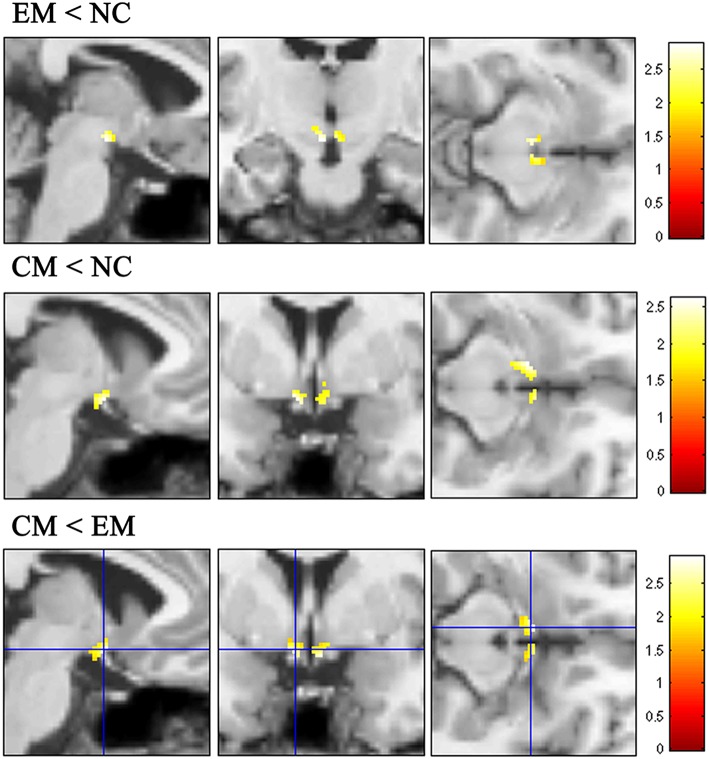
The decreased HTH volume of EM located in the posterior HTH (top line), while the decreased HTH volume of CM located in the anterior HTH (middleline) compared with NC. Compared with EM, the decreased HTH region located in the anterior HTH (bottom line).

VBM analysis identified that the decreased HTH volume of EM located in the posterior HTH (MNI coordinate:−3−12−12; 3−12−11), and the decreased HTH volume of CM located in the anterior HTH (MNI coordinate:−6 0−14; 6 3−14) compared with NC (Figure 5 and Table 2). There was no increased HTH volume of EM and CM compared with NC.

**Table 2:** The decreased HTH regions in EM and CM compared with NC.

**Group**	**Anatomic region**	**MNI-space**			**Cluster size**	***P*_**uncorr**_**	**Peak T value**
		**X**	**Y**	**Z**			
EM < NC							
	Posterior HTH	−3	−12	−12	22	0.004	2.88
	Posterior HTH	3	−12	−11	22	0.010	2.43
CM < NC							
	Anterior HTH	−6	0	−14	47	0.007	2.62
	Anterior HTH	6	3	−14	21	0.010	2.44
CM < EM							
	Anterior HTH	6	3	−14	38	0.003	2.91
	Anterior HTH	−5	3	−12	46	0.004	2.80

The decreased HTH volume of CM located in the anterior HTH (MNI coordinate:6 3−14;-5 3−12), and there was no increased HTH region in CM compared with EM (Figure 5).

### Correlation Analysis of the Volume of Anterior and Posterior HTH With the Clinical Variables

The mean decreased volume of the anterior HTH of CM and posterior HTH of EM were 0.162 ± 0.014 ml and 0.109 ± 0.009 ml. In CM patients, the volume of anterior HTH with decreased volume in VBM comparison presented positive correlation with headache frequency (*r* = 0.681, *P* = 0.001), and presented no significant correlation with the other clinical variables including VAS score, diseased duration, HAMA, HAMA and MoCA score (*P* > 0.05) (Table 3). In contrast, the volume of posterior HTH with decreased volume in VBM comparison for EM vs. NC showed significant negative correlation with headache frequency (*r* = −0.457, *P* = 0.028), and showed no significant relation with the other clinical variables including VAS score, diseased duration, HAMA, HAMA and MoCA score (*P* > 0.05).

**Table 3:** Correlation analysis between decreased HTH regions in EM and CM.

	**EM**		**CM**	
	***r***	***P*-value**	***r***	***P*-value**
VAS	0.059	0.409	0.011	0.483
DD	0.133	0.3	−0.037	0.442
HF	−0.457	0.028	0.681	0.001
HAMA	0.028	0.456	0.319	0.099
HAMD	0.279	0.131	−0.033	0.448
MoCA	0.057	0.412	−0.245	0.163

*VAS, visual analog scale; DD, disease duration; HF, headache frequency; HAMA, Hamilton Anxiety Scale; HAMD, Hamilton Depression Scale; MoCA, Montreal Cognitive Assessment*.

### Altered Functional Connectivity (FC) of Positive HTH Region According to VBM Comparison Among EM, CM, and NC

**Figure 6:**
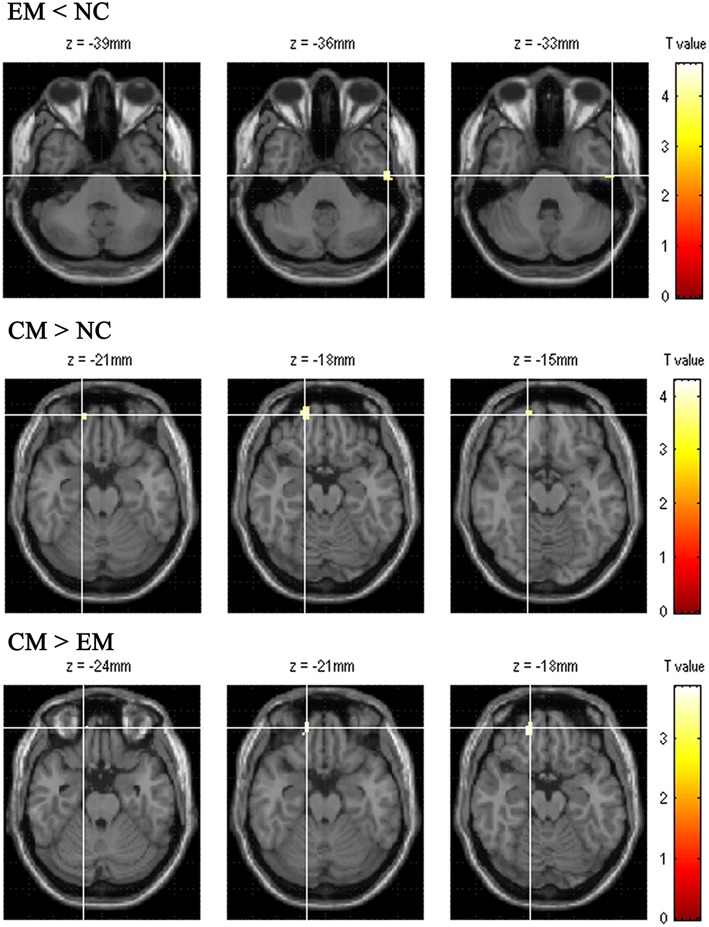
The altered functional connectivity of positive HTH region among EM, CM and NC. The top line presented the decreased functional connectivity of posterior HTH located in the left inferior temporal gyrus in EM compared with that in NC. The middle line presented the increased functional connectivity of anterior HTH of CM located in the right anterior orbital gyrus compared with NC. Bottom line showed that the increased functional connectivity of anterior HTH of CM located in the right medial orbital gyrus compared with EM.

**Figure 7:**
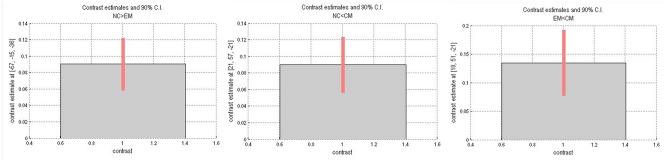
Contrast estimates and 90% CI for the significant voxels.

Table 4 presented that the decreased FC of posterior HTH located in the left inferior temporal gyrus (Brodmann area 20) in EM compared with NC, and there was no increased FC in EM compared with NC (Figure 6). The increased FC of anterior HTH of CM located in the right anterior orbital gyrus (AOrG) (Brodmann area 11) compared with NC, and the increased FC of anterior HTH of CM anchored in the right medial orbital gyrus (MOrG) (Brodmann area 11) compared with EM (Figure 6). There was no decreased FC of anterior HTH in CM compared with NC and EM. Figure 7 presented the contrast estimates for the significant voxel.

**Table 4:** The altered resting-state functional connectivity of HTH subregions with altered volume among EM, CM, and NC.

**Group**	**Brodmann** **area**	**Anatomic** **region**	**MNI-space**			**Cluster size**	***P*_**uncorr**_**	**Peak T** **value**
			**X**	**Y**	**Z**			
EM < NC								
	BA 20	Left ITG	−57	−15	−36	13	0.000	4.63
CM > NC								
	BA 11	Right AOrG	21	57	−21	16	0.000	4.29
CM > EM								
	BA11	Right MOrG	18	51	−21	24	0.000	3.86

*AOrG, anterior orbital gyrus; MOrG, medial orbital gyrus; ITG, inferior temporal gyrus*.

## Discussion

To our knowledge, this is the first study conducted to identify the changes of morphology and functional connectivity (FC) of the HTH in interictal EM and CM. In this study, both EM and CM patients had lower HTH volume than NC. The cut-off volume of HTH as 1.429 ml showed a good level for the diagnosis of CM by ROC analysis. VBM analysis further identified that the decreased HTH volume located in posterior HTH for EM and in anterior HTH for CM compared with NC. The altered volume in these HTH regions had association with headache frequency and presented altered FC with different brain regions, respectively. As a whole, our study added evidence that HTH may play an important role in migraine and migraine chronification.

HTH serves as a crucial center for the integration and coordination of various brain functions. Despite its relatively small size, the HTH expresses a large number of different neurotransmitters and peptide hormones and has wide connection to other brain regions (20). Anatomically HTH can be divided into the lateral and medial level and four rostrocaudal levels: preoptic, anterior, tuberal, and posteror HTH (21). Each region has distinct patches of nuclei and associated functions (22). The preoptic area contains medial preoptic nucleus, uncinate nucleus, intermediate nucleus, and is known to control thermoregulation, reproduction, and electrolyte balance. The anterior HTH, including the supraoptic nucleus, suprachiasmatic nucleus, paraventricular nucleus and anterior periventricular nucleus, regulates feeding, circadian rhythms, autonomic system, and other homeostatic processes. The tuberal HTH includes the arcuate nucleus, median eminence, and ventromedial and dorsomedial hypothalamus, and plays a role in energy balance, stress response, menstrual cycle, pain modulation and aggression. The posterior HTH, which includes the mammillary bodies and the dorsally located posterior hypothalamic nucleus, is involved in processing emotion, as well as spatial and episodic memory (6, 20, 21, 23).

The role of HTH in cluster headache (CH) and other trigeminal autonomic cephalalgias (TACs) has been identified in many studies. Positron emission tomography (PET) and functional MRI studies demonstrated that the posterior HTH is activated during attacks of CH and some other TACs (24, 25). Resting-state functional MRI studies also found altered FC of HTH with the salience network (SN) (26) and a number of diencephalic-mesencephalic dopaminergic structures (27) in CH. VBM MRI has found significant structural differences in the HTH posterior gray matter compared with controls (28). Considering the important role of posterior HTH in CH, deep brain stimulation (DBS) of posterior HTH has been developed as an optional treatment which produced a decrease in attack frequency of more than 50% in 60% of chronic CH patients (29). In recent years, the important role of HTH in migraine has also been revealed by functional neuroimaging studies (10–13, 30) implicating a potential therapeutic target for migraine. HTH has shown co-activation and altered FC with brainstem immediately before or during migraine attack (10–12) suggesting that hypothalamo–brainstem connectivity may be the real driver of migraine attacks. Even in interictal phase of migraine, HTH showed increased FC with a number of brain regions involved in regulation of autonomic functions, including the locus coeruleus, caudate, parahippocampal gyrus, cerebellum, and the temporal pole, which may explain some of the hypothalamic-mediated autonomic symptoms that accompany or precede migraine attacks (30). The altered HTH volume in our study further supported the role of HTH in the pathogenesis of migraine. Different from the increased HTH volume of CH (28, 31), patients of both EM and CM in our study had decreased HTH volume, indicating different neuromechanism of migraine from CH. Unlike the previous study analyzing FC of the whole HTH in interictal migraine (30), our study focused on the FC of the positive HTH regions detected in VBM comparisons and thus may further reveal the mechanism of HTH sub-regions in migraine and its chronification.

The sub-regions of HTH related to CH and migraine mainly located in posterior HTH (13, 14, 24, 25, 28) and anterior HTH (14, 31) as reported in the neuroimaging studies. The posterior HTH may be involved in headache generation (12, 13) and acute pain (14). In our study, the reduced volume of posterior HTH was only present in interictal EM and the volume reduction was negatively correlated with headache frequency in EM, suggesting that the decreased volume of posterior HTH may reflect some deficit in pain processing or modulating systems and lead patients vulnerable to migraine generation while repetitive migraine attacks may vise verse contribute to the volume regain of posterior HTH. The volume regain was considered to be related to neuronal or glial cell genesis, cell size increase, changes in cortical synaptic connectivity, neurogenic inflammation and changes in blood flow or interstitial fluids due to repetitive migraine attacks (32, 33). But the underlying mechanism of the structural alteration is yet to be elucidated. Further functional MRI in our study found decreased FC of the posterior HTH with the left inferior temporal gyrus (Brodmann area 20). The inferior temporal gyrus (ITG) is associated with visual object recognition and has been suggested as the final location of the ventral cortical visual system, which acts as a link between auditory and visual processing, perception, and memory (34). The ITG has been reported to present lower gray matter density (35), and hyperperfusion (36) in interictal migraine. The decreased FC of posterior HTH with the left ITG in EM may suggest the connection of migraine driver with silent cortical spreading depression (37) and may reflect migrainuers' hypersensitivity to visual and auditory stimuli.

One functional MRI study found a significantly stronger activation of the anterior right hypothalamus in CM compared to HC indicating that the more anterior part seemed to play an important role in migraine chronification (14). In consistency with this study, we identified that the volume of anterior HTH decreased in CM compared with both EM and NC in VBM analysis and the volume reduction was positively correlated with headache frequency in CM, which further supported the participation of anterior HTH in migraine chronification. The anterior HTH presented increased FC with the right anterior orbital gyrus (AOrG) (Brodmann area 11) and the right medial orbital gyrus (MOrG) (Brodmann area 11) compared with NC and EM, respectively. The OrG is responsible for very complex emotional and cognitive functions. Brodmann area 11 also receives olfactory and auditory information (38). Previous studies have provided neuropsychological evidence of OrG dysfunction in CM with medication overuse such as depression, dependence, impaired task performance and decision making (39–42). Neuroimaging studies identified hypometabolism (42) and volume reduction of the OrG in CM with medication overuse (43) and without medication overuse (44). The OrG has been proven in animal experiment to exert a direct influence on the anterior HTH (45). The OrG–HTH interaction has also been identified in resting-state fMRI data of human subjects (46). Therefore, the increased FC of anterior HTH and OrG in CM in our study may indicate the role of anterior HTH in the relationship of emotional and execution dysfunction with overweight, autonomic disorder and sleep problem in CM (47). However, the altered volume and FC of anterior HTH is the cause or outcome of migraine chronification is still a debate.

The HTH has an important role in pain perception. There is evidence of anatomical connections between the HTH and the trigeminal nucleus (48). More evidences confirmed the important role of the posterior HTH in regulating trigeminovascular processing through the analgesic effect by injection of opioids, or resection, or neurostimulation of this region (49). The HTH hosts many key neuropeptide systems, such as orexins, oxytocin, neuropeptide Y, and pituitary adenylate cyclase activating protein(PACAP), etc., which have been postulated to play a role in migraine pathophysiology. Potential therapeutic compounds targeting these systems may have efficacy in treating migraine (50). Oxytocin is synthesized in magnocellular neurosecretory cells in both the supraoptic and paraventricular nuclei of the anterior HTH (51). Oxytocin receptors located in numerous brain and spinal cord regions including dorsal root and calcitonin gene-related peptide (CGRP)-expressing trigeminal ganglia neurons, suggesting a role in pain modulation (52, 53). Intranasal oxytocin was associated with strong analgesic effect for CM possibly by upregulating oxytocin receptors on trigeminal neurons following inflammatory or noxious stimulation and reducing CGRP release (52). The decreased volume of anterior HTH in CM detected in our study may in part reflect structural plasticity of Oxytocin neurons and the deficit in OrG–HTH-brainstem pain modulatory system may be one of the pathogensis of CM. Both anterior and posterior HTH also contain PACAP neurons. Descending projections of PACAP neurons from the posterior, paraventricular, lateral, dorsomedial and pre-optic hypothalamic nuclei to the trigeminocervical neurons and superior salivatory nucleus, are thought to be involved in mediating cranial autonomic symptoms and dural neuro-inflammatory mechanisms of primary headache (54). Besides, PACAP was involved in control of circadian rhythms, learning, memory, and stress (54), which may also be related to migraine pathophysiology. PACAP can induce migraine-like headache (55, 56) and the PACAP system is the subject of significant interest as a potential therapeutic target for migraine (57). Researches on these HTH neuropeptide systems, the HTH structural plasticity and functional alteration may extend our knowledge to migraine pathogenesis and treatment.

Our study has some limitations. Firstly, the age and sex were not compatible among the groups. To diminish the influence on volume and FC analysis, we used age, sex and TIV as covariance. Secondarily, we did not compare the volume difference between patients suffering headache and not suffering headache because only 2 of the 16 CM patients were headache free when taking MRI scanning. Therefore, it is unknown how headache influence the HTH volume in CM. Thirdly, we did not measure the migraine-associated neuropeptide released from the HTH in the blood. Further studies may measure these peptides to better elucidate the alteration of the HTH in neuroimaging findings.

In conclusion, we provided neuroimaging evidence that the structural plasticity and FC alteration of HTH happened in interictal EM and CM. HTH volume <1.429 ml may have a good diagnostic value for CM and would be considered as a biomarker of CM. Migraine generation may be triggered by the abnormal structure and function of the posterior HTH while the anterior HTH seemed to be associated with migraine chronification. The role of the HTH in migraine pathogenesis may be due to its connection with multiple pain processing areas and the key neuropeptide systems related to migraine. Therapeutic targets toward these peptides or abnormal HTH regions as detected in this study may be tested in future studies.

## Ethics Statement

This study was carried out in accordance with the recommendations of the Ethics Committee of Chinese PLA General Hospital with written informed consent from all subjects. All subjects gave written informed consent in accordance with the Declaration of Helsinki. The protocol was approved by the Ethics Committee of Chinese PLA General Hospital.

## Author Contributions

ZC conceive and wrote the paper. XC collected clinical data and wrote part of the paper. ML collected clinial data. ZC analyzed the data. LM and SY revised the paper.

### Conflict of Interest Statement

The authors declare that the research was conducted in the absence of any commercial or financial relationships that could be construed as a potential conflict of interest.
